# Genome-Wide Scan Identifies Selection Signatures in Chinese Wagyu Cattle Using a High-Density SNP Array

**DOI:** 10.3390/ani9060296

**Published:** 2019-05-30

**Authors:** Zezhao Wang, Haoran Ma, Lei Xu, Bo Zhu, Ying Liu, Farhad Bordbar, Yan Chen, Lupei Zhang, Xue Gao, Huijiang Gao, Shengli Zhang, Lingyang Xu, Junya Li

**Affiliations:** 1 Laboratory of Molecular Biology and Bovine Breeding, Institute of Animal Sciences, Chinese Academy of Agricultural Sciences, Beijing 100193, China; wangzezhao1@163.com (Z.W.); xuleirock@163.com (L.X.); zhubo525@126.com (B.Z.); yliu2333@sina.com (Y.L.); farhadnevergiveup@yahoo.com (F.B.); chenyan0204@163.com (Y.C.); zhanglupei@caas.cn (L.Z.); gaoxue76@126.com (X.G.); gaohj111@sina.com (H.G.); 2 National Engineering Laboratory for Animal Breeding, Key Laboratory of Animal Genetics, Breeding and Reproduction, Ministry of Agriculture, College of Animal Science and Technology, China Agricultural University, Beijing 100193, China; zhangslcau@cau.edu.cn; 3 College of Animal Science and Technology, Huazhong Agricultural University, Wuhan 430070, China; mhb1971@126.com; 4 Institute of Animal Husbandry and Veterinary Research, Anhui Academy of Agricultural Sciences, Hefei 230031, China

**Keywords:** Chinese Wagyu cattle, economic trait, selection signatures, high-density SNP array

## Abstract

**Simple Summary:**

Analysis of the genomic regions under consideration can provide important insights into the genetic basis of complex traits. In this study, we utilized a high-density SNP (Single nucleotide polymorphism) array for analysis of genome selection signatures in Chinese Wagyu cattle. In total, 239 selected regions responding to 162 candidate genes were identified, which related to economic traits, including fatty acid, meat quality, growth and developmental traits. Moreover, we found that these selected genes were enriched for the four Gene Ontology (GO) terms of biological regulation and metabolic process. These results also contribute to the understanding of genetic basis of these traits during the formation of this population.

**Abstract:**

Selective breeding can lead to genetic diversity and diverse phenotypes in farm animals. Analysis of the genomic regions under selection can provide important insights into the genetic basis of complex traits. In this study, a high-density SNP array was used for analysis of genome selection signatures in Chinese Wagyu cattle. In total, we obtained 478,903 SNPs and 24,820 no-overlap regions for |iHS| (integrated haplotype score) estimations. Under the threshold of the top 1%, 239 regions were finally identified as candidate selected regions and 162 candidate genes were found based on the UMD3.1 genome assembly. These genes were reported to be associated with fatty acids, such as Bos taurus nitric oxide synthase 1 adaptor protein (*NOS1AP*), Bos taurus hydroxysteroid 17-beta dehydrogenase 7 (*HSD17B7*), Bos taurus WD repeat domain 7 (WDR7), Bos taurus ELOVL fatty acid elongase 2 (*ELOVL2*), Bos taurus calpain 1 (*CAPN1*), Bos taurus parkin RBR E3 ubiquitin protein ligase (*PRKN*, also known as *PARK2*), Bos taurus mitogen-activated protein kinase kinase 6 (*MAP2K6*), meat quality, including Bos taurus ADAM metallopeptidase domain 12 (*ADAM12*), Bos taurus 5′-aminolevulinate synthase 1 (*ALAS1*), Bos taurus small integral membrane protein 13 (*SMIM13*) and Bos taurus potassium two pore domain channel subfamily K member 2 (*KCNK2*), growth, and developmental traits, such as Bos taurus insulin like growth factor 2 receptor (*IGF2R*), Bos taurus RAR related orphan receptor A (*RORA*), Bos taurus fibroblast growth factor 14 (*FGF14*), Bos taurus paired box 6 (*PAX6*) and Bos taurus LIM homeobox 6 (*LHX6*). In addition, we identified several genes that are associated with body size and weight, including Bos taurus sorting nexin 29 (*SNX29*), Bos taurus zinc finger imprinted 2 (*ZIM2*), Bos taurus family with sequence similarity 110 member A (FAM110A), immune system, including Bos taurus toll like receptor 9 (*TLR9*), Bos taurus TAFA chemokine like family member 1 (*TAFA1*), Bos taurus glutathione peroxidase 8 (putative) (*GPX8*), Bos taurus interleukin 5 (*IL5*), Bos taurus PR domain containing 9 (*PRDM9*), Bos taurus glutamate ionotropic receptor kainate type subunit 2 (*GRIK2*) and feed intake efficiency, Bos taurus sodium voltage-gated channel alpha subunit 9 (*SCN9A*), Bos taurus relaxin family peptide/INSL5 receptor 4 (*RXFP4*), Bos taurus RNA polymerase II associated protein 3 (*RPAP3*). Moreover, four GO terms of biological regulation (GO:0009987, GO:0008152) and metabolic process (GO:0003824, GO:0005488) were found based on these genes. In addition, we found that 232 candidate regions (~18 Mb) overlapped with the Quantitative trait loci (QTL)regions extracted from cattle QTLdb. Our findings imply that many genes were selected for important traits in Chinese Wagyu cattle. Moreover, these results can contribute to the understanding of the genetic basis of the studied traits during the formation of this population.

## 1. Introduction

Selection in cattle has generated divergent breeds that are specialized for various characteristics, which also results in selection signatures that relate to important traits across genomes. Under positive selection pressure, the frequency of favorable alleles and particular regions for relevant traits have rapidly increased [1]. This may also have an impact on haplotype structure and cause extended linkage disequilibrium (LD) patterns between the mutation and neighboring loci. Thus, analysis of genomic regions under selection can provide important insights into the genetic basis of complex traits [2].

Many recent studies have been conducted for the detection of signals of recent positive selections using various statistical approaches, including extended haplotype homozygosity (EHH) [3], integrated haplotype score (iHS) [4], cross-population Extended Haplotype Homozygosity (XPEHH) [5], and nSL [6]. The EHH approach is particularly proposed to detect signatures of positive selections within populations based on single nucleotide polymorphisms (SNPs) [7,8], which can identify genomic regions with extended haplotypes within populations. To avoid the influence of heterogeneous recombination rates across the genome, Voight et al. [4] proposed the “integrated Haplotype Score” (iHS) approach based on an extension of the EHH method. The iHS method is most suitable for detecting breed-specific genes under positive selection [9,10]. The iHS method can identify selection signatures in the regions with strong LD surrounding the selected site. Many studies have been conducted in human [4,11] and pig populations [12], as well as in cattle populations, using iHS method [7,13,14,15,16]. 

In cattle, previous studies have been conducted to explore the genome selection signatures using both allele frequency [1,7,17,18] and extended haplotype methods [4,7,19,20]. The allele frequency-based method has the power to detect the loci with population differences, while the extended haplotype approach was proposed to identity recent breed-specific selection events [4]. Several studies involving genome association and genetic parameter estimation of important traits in Wagyu have been conducted [21,22,23,24]. However, selection signatures for this breed and other related populations are yet to be explored.

Chinese Wagyu cattle is a hybrid population from Wagyu and Fuzhou cattle, while Fuzhou cattle is an indigenous population raised in Liaoning Province, China. Wagyu cattle is a highly selective breed that has been developed over the past decades. It is especially well-known for its remarkable marbling score [21,25]. Because the marbling is mainly fat tissue containing abundant monounsaturated fatty acids, it contributes to beef flavor and tenderness [21,26]. Investigation of the genetic basis of important traits can provide valuable insights to the design of breeding strategies and further offer more economic benefits in the beef market. 

The objectives of our study were to (1) detect signatures of the selection using a high-density SNP array on Chinese Wagyu cattle, (2) identify the selected regions and their candidate genes for economic important traits, and (3) explore the potential biological functions of the candidate genes under selection using bioinformatics analyses.

## 2. Material and Methods 

### 2.1. Ethics Statement

No ethics statement was required for the collection of genetic material. The dataset from animals included in this study were from previous analyses that obtained specific permissions [27].

### 2.2. Genotypes

The Chinese Wagyu population (464 animals) were genotyped using the Illumina Bovine HD BeadChip (770K, Illumina, Inc., San Diego, CA, USA). The data were extracted from our pervious publication [27]. SNPs were pre-processed based on the following filters using PLINK v1.07 (Broad Institute of Harvard and MIT, Cambridge, MA, USA): proportion of missing genotypes (<0.05), Minor allele frequency (<0.05) and Hardy-Weinberg equilibrium (*p* < 10^−6^). Moreover, individuals with more than 10% missing genotypes were excluded. Missing genotypes were imputed, and genotypes were phased, using Beagle v3.3.2 (University of Washington, St. Louis, MO, USA). After quality control, the final data consisted of 364 individuals and 503,537 autosomal SNPs. The mean distance between adjacent SNPs was 4.99 kb.

### 2.3. Detection of Selection Signatures Using iHS

The iHS score was estimated for each autosomal SNPs using the selscan program with default settings. This tool is ‘dumb’ with respect ancestral/derived coding and simply expects haplotype data to be coded 0/1. Unstandardized iHS scores are reported as ln(iHH1/iHH0) based on the coding provided [28].

The normal standardized iHS was calculated as

$$\mathrm{iHS}=\frac{\ln\left(\frac{iHH_{A}}{iHH_{D}}\right)-E_{p}[ln\left(\frac{iHH_{A}}{iHH_{D}}\right)]}{SD_{p}[\ln\left(\frac{iHH_{A}}{iHH_{D}}\right)]}$$

where $iHH_{A}$ and $iHH_{D}$ represent the integrated EHH score for ancestral and derived core alleles, respectively. $E_{p}[ln\left(\frac{iHH_{A}}{iHH_{D}}\right)]$ and $SD_{p}[\ln\left(\frac{iHH_{A}}{iHH_{D}}\right)]$ are the expectation and standard deviation in frequency bin p. Single-site iHS values were computed across the genome in the Chinese Wagyu population and averaged within non-overlapping windows of 100 kb across the genome. The sites with |iHS| scores higher than 2 (the top 1%) were considered to be putative signatures of selection [29]. The top 1% regions with the highest average |iHS| scores were used in the downstream analyses, and the windows with SNP number <10 were dropped in our analysis [4,30]. 

### 2.4. Bioinformatics Analyses of Candidate Genes Under Selection

For the iHS test, we only considered genes within the top 1% regions. Genes in the candidate regions were retrieved by exploiting the knowledge on Cow Nov. 2009 (Bos_taurus_ UMD 3.1/bosTau6) Assembly from the University of California Santa Cruz (UCSC) Brower [31]. Moreover, to explore the biological functions and pathways of selected genes, we used the Database for Annotation, Visualization, and Integrated Discovery (DAVID; version 6.8, Leidos Biomedical Research, Inc., Frederick, MD, USA) tool for gene enrichment analysis [32]. Significant GO terms provide insight into the functional characteristics of annotated genes. The Kyoto Encyclopedia of Genes and Genomes (KEGG) database (Kyoto University, Uji, Kyoto, Japan) was also cross-referenced within DAVID to identify significant pathways. GO terms involved in molecular functions, biological processes, and cellular components were selected as the functional annotation category in our studies. To better understand the molecular functions of these genes, we examined their GO classifications. Also, we investigated the network using the PANTHER v14.0 classification system (University of Southern California, Los Angeles, CA, USA). Based on 496 genes, we tested the hypothesis that the PANTHER molecular function, biological process, and pathway terms were under- or over-represented in genes regions after Bonferroni corrections [33]. To detect whether the selective genes overlap with the QTLs Associated with important traits, we downloaded QTL information from the cattle QTLdb at http://www.animalgenome.org/cgi-bin/QTLdb/BT/index (last accessed 2 November 2018). Because previous QTL mapping studies have utilized various markers and employed different design populations and mapping methods, we merged all QTLs into a set of unique non-redundant regions.

## 3. Results 

### 3.1. Detection of Selection Signature Using an iHS Approach

**Figure 1:**
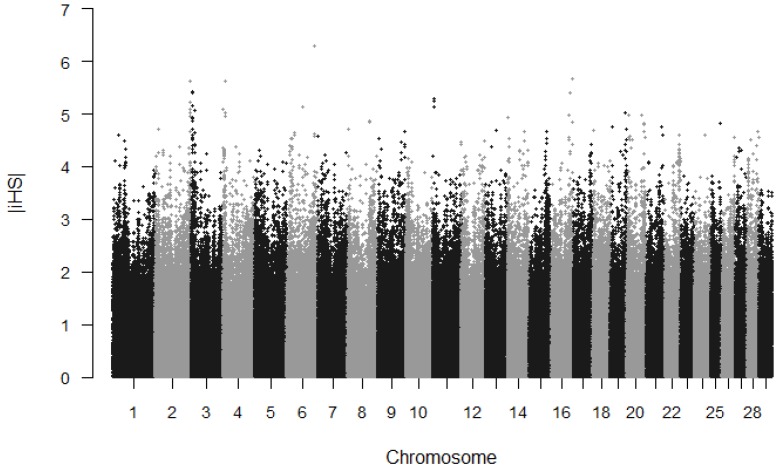
Genome-wide distribution of integrated haplotype score (|iHS|) values estimated in Chinese Wagyu cattle.

In this study, we utilized 503,537 high quality SNPs for analysis of a genome selection signature in Chinese Wagyu cattle, these SNPs covered 2.5 Gb of the cattle genome (UMD3.1), with an average distance of 4.99 kb between adjacent SNPs. The summary statistics’ average distance between the adjacent SNPs across the autosome is presented in supplementary Table S1. We investigated potential evidence of recent positive selections based on the |iHS| score. The value of the iHS score per site was calculated and then averaged in non-overlapping 100 k regions across the genome. We obtained a total 478,903 SNPs with estimated |iHS| scores (supplementary Table S2). All of SNP sites were normalized and then used for the identification of candidate regions. As shown in Figure 1, the genome-wide distribution of |iHS| values was generated to visualize the chromosomal distribution of selection signatures. in total, 24,820 regions were detected based on the single site |iHS| score. Under the threshold of the top 1%, we finally identified 239 regions as candidate regions. The highest estimated value is 2.502 for the identified window located on BTA3, containing 38 SNPs (supplementary Table S3). The top 20 significant |iHS| genomic regions and candidate genes are shown in Table 1.

### 3.2. Genes and QTLs Based on Identified Regions

We investigated the genomic regions containing extreme |iHS| scores using UMD 3.1 from the UCSC Brower. In total, 162 candidate genes overlapped with significant iHS genomic regions detected in the studied population (supplementary Table S4). Moreover, we investigated the regions overlapped with the QTL region extracted from the QTLdb for cattle. We found that 232 candidate regions (~18 Mb) overlapped with the merged QTL regions. Most of regions that overlapped with the QTL region were found to be related to economically important traits in cattle. 

### 3.3. Functional Classification based on Selection Genes

**Figure 2:**
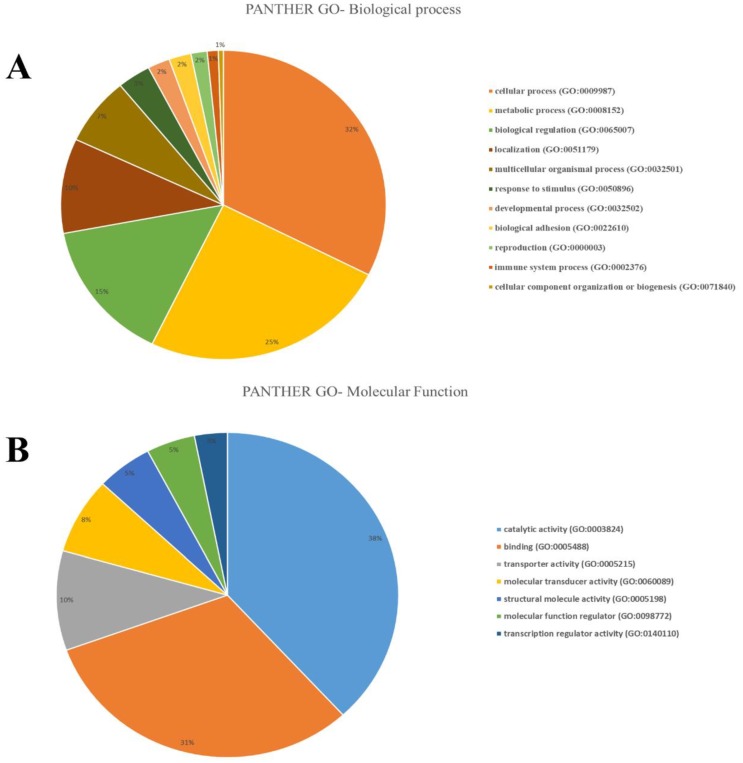
(**A**) The PANTHER GO-Biological process based on selection genes, showing the percent of gene hit against total # Process hits. (**B**) The PANTHER GO-Molecular Function based on selection genes, showing the percent of gene hit against total # Function hits.

Using the DAVID tool, we explored the biological functions and pathways of 162 selected genes. However, no significant GO term for the selected molecular functions, biological processes, and cellular components was found based on the queried genes. Using the PANTHER classification system, we found that these selected genes were enriched for the four GO terms of biological regulation (GO:0009987, GO:0008152), and the metabolic process (GO:0003824, GO:0005488) (Figure 2). 

### 3.4. Candidate Genes under Selection

Table 1 summarizes the candidate genes that overlapped with the top 20 significant iHS regions. We observed that many identified regions overlapped with genes that have been previously reported under selection. Some genes were related to fatty acids, such as Bos taurus nitric oxide synthase 1 adaptor protein (*NOS1AP*), Bos taurus hydroxysteroid 17-beta dehydrogenase 7 (*HSD17B7*), Bos taurus WD repeat domain 7 (*WDR7*), Bos taurus ELOVL fatty acid elongase 2 (*ELOVL2*), Bos taurus calpain 1 (*CAPN1*), Bos taurus parkin RBR E3 ubiquitin protein ligase (*PRKN*, also known as *PARK2*), Bos taurus mitogen-activated protein kinase kinase 6 (*MAP2K6*), meat quality, including Bos taurus ADAM metallopeptidase domain 12 (*ADAM12*), Bos taurus 5′-aminolevulinate synthase 1 (*ALAS1*), Bos taurus small integral membrane protein 13 (*SMIM13*) and Bos taurus potassium two pore domain channel subfamily K member 2 (*KCNK2*), growth, and developmental traits, such as Bos taurus insulin like growth factor 2 receptor (*IGF2R*), Bos taurus RAR related orphan receptor A (*RORA*), Bos taurus fibroblast growth factor 14 (*FGF14*), Bos taurus paired box 6 (*PAX6*) and Bos taurus LIM homeobox 6 (*LHX6*). In addition, we identified several genes that are associated with body size and weight, including Bos taurus sorting nexin 29 (*SNX29*), Bos taurus zinc finger imprinted 2 (*ZIM2*), Bos taurus family with sequence similarity 110 member A (*FAM110A*), immune system, including Bos taurus toll like receptor 9 (*TLR9*), Bos taurus TAFA chemokine like family member 1 (*TAFA1*), Bos taurus glutathione peroxidase 8 (putative) (*GPX8*), Bos taurus interleukin 5 (*IL5*), Bos taurus PR domain containing 9 (*PRDM9*), Bos taurus glutamate ionotropic receptor kainate type subunit 2 (*GRIK2*) and feed intake efficiency, Bos taurus sodium voltage-gated channel alpha subunit 9 (*SCN9A*), Bos taurus relaxin family peptide/INSL5 receptor 4 (*RXFP4*), Bos taurus RNA polymerase II associated protein 3 (*RPAP3*). 

## 4. Discussion

The phenotypic variations among the population are caused by both natural selection and artificial selection in farm animals [34]. Identifying positive selection signatures can provide valuable information about the influence of selection pressures for economically important traits. This strategy can act as a complement for current gene mapping approaches and further help to elucidate the genetic basis of complex traits. We have attempted to detect recent selection signatures in the Chinese Wagyu cattle population using a high-density genotype array. The average distance between adjacent SNPs was 4.99 kb, which is smaller than the previous reports using a low-density SNP array [7,14]. The application of a high-density SNP chip for the detection of genomic selection signatures is more efficient to identify core haplotypes across genomes [14]. 

### 4.1. Candidate Genes Related to Meat Quality

Chinese Wagyu derived from Wagyu cattle, that is especially well-known for its high meat quality [21,25]. Previous studies suggested that the marbling score was selected in Wagyu cattle [21,35,36]. Thus, we suspected that the identified candidate genes under recent selection are likely to be related to marbling and fatty acids. In this study, we detected 17 genes for the remarkable marbling score and meat quality. For instance, we found one region at 45.9–46 Mb on Bos taurus chromosome 26th (BTA26) with a high average |iHS| value (2.162). This region contains the *ADAM12* gene, which may be involved in the regulation of myogenesis and adipogenesis in beef cattle [37,38]. Also, this gene was identified as a regulator for TGF-β1 [38]. The transforming growth factor-β1 (TGF-β1) can induce the differentiation of human adipose tissue-derived mesenchymal stem cells into smooth muscle cells. 

We also detected one candidate region located at 44–44.1 Mb on Bos taurus chromosome 29th (BTA29), which contains the gene *CAPN1*. *CAPN1* was reported to code for proteolytic enzymes and regulates the rate of proteolytic changes in cells [39]. This gene is responsible for the protein breakdown in meat post-mortem and associated with high free fatty acid levels [40]. *CAPN1* also has a significant relationship with the marbling score and meat maturation [39,41]. In addition, several candidate markers that relate to meat tenderness in the *CAPN1* gene have been previously reported [42,43,44,45,46], as well as meat color [47] and pH [48] in various cattle breeds. Recent studies have shown that several polymorphisms near *CAPN1* were significantly associated with Warner–Bratzler shear force in Nelore beef cattle [49]. These results suggested that a desirable meat quality phenotype might result from a specific haplotype structure containing alleles of calpain genes, which had been formed during long-term selection. [50,51]. 

### 4.2. Genes Related to Fatty Acids

Our study identified two genes, *ELOVL2* and *AANAT*, as potential candidate under recent selection. Significant evidence has suggested that these genes are related to fatty acid traits. For instance, the percentages of C16:0, C16:1cis-9 and C20:3n-9 showed positive correlations with the expression levels of *ELOVL2*. A negative correlation was observed between the mRNA level of *ELOVL2* and the percentage of C17:0 [52]. Three genes including *HSD17B7*, *WDR7*, and *MAP2K6* have been previously detected as related to the fatty acid profile in Nellore cattle [53]. Our results further suggested that potential selection may act on the fatty acid profile in Chinese Wagyu cattle. Many previous studies showed that *AANAT* affects several meat quality traits, such as omega-3 Sum of polyunsaturated fatty acid (PUFA), fatty aldehyde, and collagen muscle content, flavor, and pH on thawed samples at 10 days postmortem in cattle [37,54,55]. However, in our study, one region with AANAT showed a relatively low mean |iHS| value (0.74), which may indicate that no genomic selection signature occurred for this region in Chinese Wagyu cattle. Also, *HSPBP1* was not detected in the current study. This gene has been reported to be associated with the tenderness of beef cattle [56] and has a considerable effect on the percentage of lauric acid (C12:0) and phospholipid docosahexaenoic acid (DHA C22:6 n-3) [40]. 

### 4.3. Genes Related to Growth and Developmental Performance

Growth and developmental performance are highly important economic traits in the beef cattle industry. Three genes related to the growth and developmental performance were identified under selection in Chinese Wagyu cattle. We found that one region at 97.6–97.7 Mb on BTA9 overlapped with *IGF2R*, which spans ∼101 kb on BTA9. This gene is considered a major inhibitor of growth, encodes a transmembrane protein receptor, and degrade the excess circulating insulin-like growth factor 2 (*IGF-II*). In addition, the *IGF2R* may serve as a candidate gene for body and growth-related traits in farm animals [57]. Previous studies have reported several candidate genes, including *PLAG1, GDF5,* and *CDK6* for body size, which have been reported in horses [58], humans [9,59], dogs [60], and other cattle populations [61]. However, these genes were not identified in the current study. This is probably due to the different genetic architecture among the breeds, while the sample size, marker densities, and method may also affect the detection of the selection signature.

## 5. Conclusions

Our analyses revealed multiple genes, related to fatty acids, body size, and muscle formation, under positive selection in Chinese Wagyu cattle. These results imply that one should select for important traits during the formation of this population. Our findings contribute to the identification of candidate genes for the underlying important traits in Chinese Wagyu cattle. 

## Figures and Tables

**Table 1:** Genomic region and associated genes of the top 20 significant |iHS| ^3^.

BTA ^1^	Position (Mbp)	NO. of SNP ^2^ (n)	Mean |iHS| Value	Candidate Genes	Gene Name
2	30.1–30.2	22	2.198	*SCN9A*	Bos taurus sodium voltage-gated channel alpha subunit 9
2	134.4–134.5	34	2.098	*TAS1R2*	Bos taurus taste 1 receptor member 2
3	14.8–14.9	38	2.502	*ARHGEF2*	Bos taurus Rho/Rac guanine nucleotide exchange factor (GEF) 2
3	14.8–14.9	38	2.502	*KIAA0907*	Bos taurus KH domain containing 4, pre-mRNA splicing factor
3	14.8–14.9	38	2.502	*RXFP4*	Bos taurus relaxin family peptide/INSL5 receptor 4
3	14.8–14.9	38	2.502	*SSR2*	Bos taurus signal sequence receptor subunit 2
3	7.2–7.3	40	2.298	*NOS1AP*	Bos taurus nitric oxide synthase 1 adaptor protein
3	6.6–6.7	32	2.119	*DDR2*	Bos taurus discoidin domain receptor tyrosine kinase 2
3	6.6–6.7	32	2.119	*HSD17B7*	Bos taurus hydroxysteroid 17-beta dehydrogenase 7
5	69.2–69.3	16	2.332	*C5H12orf75*	Bos taurus chromosome 5 C12orf75 homolog
5	37.3–37.4	16	2.261	*ADAMTS20*	Bos taurus ADAM metallopeptidase with thrombospondin type 1 motif 20
5	29.1–29.2	19	2.126	*METTL7A*	Bos taurus methyltransferase like 7A
5	28.3–28.4	38	1.982	*SCN8A*	Bos taurus sodium voltage-gated channel alpha subunit 8
9	97.6–97.7	13	2.049	*AIRN*	Bos taurus antisense IGF2R RNA noncoding
9	97.6–97.7	13	2.049	*IGF2R*	Bos taurus insulin-like growth factor 2 receptor
10	11.5–11.6	42	2.415	*TXNDC16*	Bos taurus thioredoxin domain containing 16
10	11.4–11.5	28	2.05	*ERO1A*	Bos taurus endoplasmic reticulum oxidoreductase 1 alpha
10	11.4–11.5	28	2.05	*PSMC6*	Bos taurus proteasome 26S subunit, ATPase 6
10	10.4–10.5	22	1.985	*HOMER1*	Bos taurus homer scaffold protein 1
11	81.1–81.2	14	2.374	*VSNL1*	Bos taurus visinin like 1
13	60.7–60.8	14	1.945	*ANGPT4*	Bos taurus angiopoietin 4
13	60.7–60.8	14	1.945	*FAM110A*	Bos taurus family with sequence similarity 110 member A
17	63.5–63.6	39	2.169	*SLC8B1*	Bos taurus solute carrier family 8 member B1
22	49.2–49.3	13	1.958	*ALAS1*	Bos taurus 5′-aminolevulinate synthase 1
22	49.2–49.3	13	1.958	*POC1A*	Bos taurus POC1 centriolar protein A
22	49.2–49.3	13	1.958	*TLR9*	Bos taurus toll like receptor 9
22	49.2–49.3	13	1.958	*TWF2*	Bos taurus twinfilin actin binding protein 2
22	49.2–49.3	13	1.958	*WDR82*	Bos taurus WD repeat domain 82
26	45.9–46	30	2.162	*ADAM12*	Bos taurus ADAM metallopeptidase domain 12
28	33.5–33.6	26	2.277	*KCNMA1*	Bos taurus potassium calcium-activated channel subfamily M alpha 1
28	3.7–3.8	17	2.126	*ARV1*	Bos taurus ARV1 homolog, fatty acid homeostasis modulator

^1^ BTA: Bos taurus autosome, ^2^ SNP: Single nucleotide polymorphism, ^3^ |iHS|: the absolute value of integrated Haplotype Score.

## Supplementary Materials

The following are available online at https://www.mdpi.com/2076-2615/9/6/296/s1, Table S1: The mean distance between adjacent SNPs per chromosome, Table S2: iHS score for each SNP, Table S3: Top 1% selected region, Table S4: Genes annotation for core regions detected by iHS.
