# Supplementary material for: Genome-Wide Scan Identifies Selection Signatures in Chinese Wagyu Cattle Using a High-Density SNP Array
Source: Animals (Basel). 2019 May 30;9(6):296. doi: 10.3390/ani9060296 (PMC6617538; doi:10.3390/ani9060296)
Supplement: Supplementary file 1 [file animals-09-00296-s001.zip › Supplementary file/TableS1.docx]

| Chr | Length(Mb) | No. of SNP | Mean Distance (kb) |
| --- | --- | --- | --- |
| chr1 | 158.34 | 29609 | 5.35 |
| chr2 | 137.06 | 27605 | 4.97 |
| chr3 | 121.43 | 24228 | 5.01 |
| chr4 | 120.83 | 23319 | 5.18 |
| chr5 | 121.19 | 24851 | 4.88 |
| chr6 | 119.46 | 23575 | 5.07 |
| chr7 | 112.64 | 23203 | 4.85 |
| chr8 | 113.38 | 18111 | 6.26 |
| chr9 | 105.71 | 21682 | 4.88 |
| chr10 | 104.31 | 23005 | 4.53 |
| chr11 | 107.31 | 22813 | 4.7 |
| chr12 | 91.16 | 17089 | 5.33 |
| chr13 | 84.24 | 15005 | 5.61 |
| chr14 | 84.65 | 15092 | 5.61 |
| chr15 | 85.3 | 17163 | 4.97 |
| chr16 | 81.72 | 17164 | 4.76 |
| chr17 | 75.16 | 16326 | 4.6 |
| chr18 | 66 | 14426 | 4.58 |
| chr19 | 64.06 | 12999 | 4.93 |
| chr20 | 72.04 | 15626 | 4.61 |
| chr21 | 71.6 | 13727 | 5.22 |
| chr22 | 61.44 | 12135 | 5.06 |
| chr23 | 52.53 | 11277 | 4.66 |
| chr24 | 62.71 | 12971 | 4.84 |
| chr25 | 42.9 | 9586 | 4.48 |
| chr26 | 51.68 | 11377 | 4.54 |
| chr27 | 45.41 | 9888 | 4.59 |
| chr28 | 46.31 | 9010 | 5.14 |
| chr29 | 51.51 | 10675 | 4.82 |
| Total | 2512.08 | 503537 | 4.99 |

Table S1 The mean distance between adjacent SNPs per chromosome
